# *Piriformospora indica*: Potential and Significance in Plant Stress Tolerance

**DOI:** 10.3389/fmicb.2016.00332

**Published:** 2016-03-22

**Authors:** Sarvajeet S. Gill, Ritu Gill, Dipesh K. Trivedi, Naser A. Anjum, Krishna K. Sharma, Mohammed W. Ansari, Abid A. Ansari, Atul K. Johri, Ram Prasad, Eduarda Pereira, Ajit Varma, Narendra Tuteja

**Affiliations:** ^1^Stress Physiology and Molecular Biology Laboratory, Centre for Biotechnology, Maharshi Dayanand UniversityRohtak, India; ^2^Plant Molecular Biology Group, International Centre for Genetic Engineering and BiotechnologyNew Delhi, India; ^3^Centre for Environmental and Marine Studies and Department of Chemistry, University of AveiroAveiro, Portugal; ^4^Department of Microbiology, Maharshi Dayanand UniversityRohtak, India; ^5^Department of Biology, University of TabukTabuk, Saudi Arabia; ^6^School of Life Sciences, Jawaharlal Nehru UniversityNew Delhi, India; ^7^Amity Institute of Microbial Technology, Amity UniversityNoida, India

**Keywords:** *Piriformospora indica*, colonization potential, Ca^2+^ signaling, crop improvement, plant stress tolerance

## Abstract

Owing to its exceptional ability to efficiently promote plant growth, protection and stress tolerance, a mycorrhiza like endophytic Agaricomycetes fungus *Piriformospora indica* has received a great attention over the last few decades. *P. indica* is an axenically cultiviable fungus which exhibits its versatility for colonizing/hosting a broad range of plant species through directly manipulating plant hormone-signaling pathway during the course of mutualism. *P. indica*-root colonization leads to a better plant performance in all respect, including enhanced root proliferation by indole-3-acetic acid production which in turn results into better nutrient-acquisition and subsequently to improved crop growth and productivity. Additionally, *P. indica* can induce both local and systemic resistance to fungal and viral plant diseases through signal transduction. *P. indica*-mediated stimulation in antioxidant defense system components and expressing stress-related genes can confer crop/plant stress tolerance. Therefore, *P. indica* can biotize micropropagated plantlets and also help these plants to overcome transplantation shock. Nevertheless, it can also be involved in a more complex symbiotic relationship, such as tripartite symbiosis and can enhance population dynamic of plant growth promoting rhizobacteria. In brief, *P. indica* can be utilized as a plant promoter, bio-fertilizer, bioprotector, bioregulator, and biotization agent. The outcome of the recent literature appraised herein will help us to understand the physiological and molecular bases of mechanisms underlying *P. indica*-crop plant mutual relationship. Together, the discussion will be functional to comprehend the usefulness of crop plant-*P. indica* association in both achieving new insights into crop protection/improvement as well as in sustainable agriculture production.

## Introduction

In natural ecosystems, a variety of microorganisms seek to obtain nutrients for their survival by interacting with plants, where the interaction can be neutral, harmful (parasitism), or beneficial (mutualism or symbiosis) to the host (Shen et al., 2006; Thrall et al., 2007). However, most plants in natural ecosystems have been reported to display their high degree of colonization/symbiosis with mycorrhizal fungi and/or fungal endophytes (Rodriguez et al., 2009; Zuccaro et al., 2011). Biotrophy, necrotrophy and hemibiotrophy are among the major lifestyles that plant-associated fungi can exhibit (Zuccaro et al., 2011). The actively metabolizing plant tissues are required by biotrophic fungi where host is kept alive; whereas, the host is killed by necrotrophic fungi to obtain nutrients from the dead cells for their own growth and survival. The hemibiotrophic fungi belongs to an intermediate category, which requires living host cells during the initial part of their life cycles, and later acts as a necrotrophic fungi (Zuccaro et al., 2011).

*Piriformospora indica*, an axenically cultivable phytopromotional, biotrophic mutualistic root endosymbiont belongs to order Sebacinales (Basidiomycota) and has been reported to mimic capabilities of typical arbuscular mycorrhizal (AM) fungi. This fungus can colonize roots of a wide range of higher plants and provide plants multifaceted amenities (such as nutrient uptake, disease resistance, stress tolerance and growth-promotion involving value addition) (Unnikumar et al., 2013). In plant groups other than crops, for example orchids, *P. indica* has been reported to be primarily existed as a partner of mycorrhiza (Schäfer and Kogel, 2009). This fungus has been reported to perform multifarious functions, including its role in biological hardening during transplantation of micro-propagated plantlets (Singh et al., 2003), increased endogenous content of spilanthol after realization of its mutual interaction with medicinal plants such as *Spilanthes calva* (Rai et al., 2004). *P. indica* infestation in *Helianthus annus* and *Aristolochia elegans* has resulted into the stimulated synthesis of valuable compounds (Bagde et al., 2010a, 2014). Additionally, *Bacopa monnieri* co-cultivated with *P. indica* exhibited an enhanced growth, elevated bacoside endogenous level, antioxidant activity and nuclear hypertrophy (Prasad et al., 2013). Notably, compared to many other endophytes, *P. indica* can be cultured very easily in a bioreactor in order to prepare effective biofertilizer formulations (Singh et al., 2003; Oelmüller et al., 2009; Bagde et al., 2010b; Qiang et al., 2011). *P. indica* inocula are very effective for their commercial applications to various crops within the defined parameters *viz.,* inocula quantity, inoculation time point, as well as soil selection for plant cultivation. Moreover, *P. indica* root endophyte has been credibly evidenced to minimize the use of chemical fertilizers, control crop yield, and also to provide increased resistance and tolerance in plants against biotic and abiotic stresses (Unnikumar et al., 2013). In our recent effort, *P. indica*-mediated improvements in the biomass, seed germination, plant growth and development and crop productivity under favorable environmental conditions were highlighted, and *P. indica* was argued as a powerful tool for crop improvement (Ansari et al., 2014).

Taking into account of recent literature, this paper: (a) overviews *P. indica*-strategies for root colonization; (b) gives insights into *P. indica*–plant mutualistic interaction and the role of calcium; (c) enlightens the association of *P. indica* with programmed cell death (PCD); (d) dissects information related with *P. indica* genome; (e) appraises literature available on *P. indica*-services to plants; (f) evaluates interaction of *P. indica* with other microorganisms, and appraises biotechnological significance of *P. indica*; (g) cross-talks information related with regulatory role of *P. indica* for the genes involved in plant metabolism, mineral uptake, in plant stress resistance and defense; and finally, (h) highlights the least explored aspects in the present context so far.

## *Piriformospora indica*–Strategies for Root-Colonization

**FIGURE 1:**
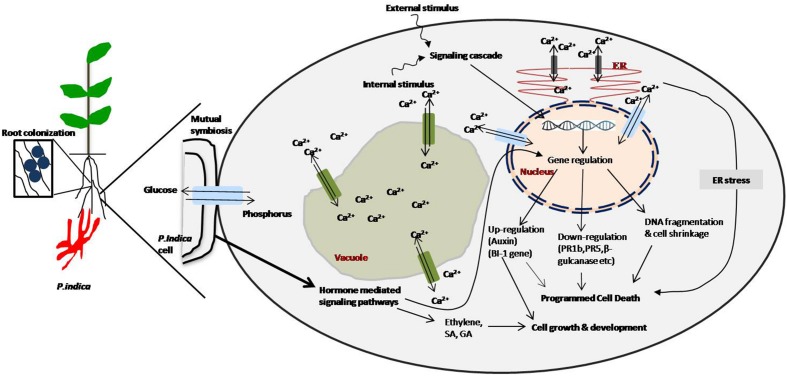
**Schematic representation of cellular and biochemical signaling cascades mediated by *Piriformospora indica* and plant colonization.** Calcium signaling is induced by signals: Ca^2+^ eﬄux and influx homeostasis in vacuole, endoplasmic reticulum (ER) and nucleus by transporter, programmed cell death (PCD) induced by external and internal stimuli generated after *P. indica* colonization. PCD may be induced by genomic fragmentation and up and down regulation of few genes. PCD is also mediated by ER stress. Growth and development can be mediated by auxin, salicylic acid (SA) and ethylene phytohormones production and immune responses generated by gibberelic acid (GA) and jasmonic acid (JA) hormones-signaling (Hückelhoven, 2004; Lam, 2004; Deshmukh et al., 2006; Deshmukh and Kogel, 2007; Schäfer et al., 2009; Vadassery and Oelmüller, 2009; Jacobs et al., 2011; Qiang et al., 2011).

The fact that *P. indica* has a broad host range, which is not only confined to vascular plants but also to colonized mosses, implies that this fungus has evolved highly effective colonization strategies (Qiang et al., 2011). Some facts related with the lifestyle and the mechanisms underlying root colonization of *P. indica* from its interaction with many plants (such as *Hordeum vulgare* and *Arabidopsis thaliana*) have been unraveled (Deshmukh et al., 2006; Schäfer and Kogel, 2009). Generally, symbionts colonize rhizodermal and cortical cell layers of roots (Khatabi, 2009). Root colonization by *P. indica* is also known to start with interacellular chlamydospore germination and forming extracellular hyphal mats, and simultaneously penetrating rhizodermal and cortical cells (Deshmukh et al., 2006; Jacobs et al., 2011). As colonization proceeds, roots are densely covered with extracellular hyphae and harbor through inter- and intracellular networks; however, the fungus never enters into the vascular tissues. At cellular level, this fungus colonizes living root cells by its direct penetration (Jacobs et al., 2011). No microscopic evidence for impairment or even necrotization was found in *H. vulgare* and *P. indica-colonized A. thaliana* roots (Schäfer and Kogel, 2009). The colonization patterns of the various root regions harbor some quantitative as well as qualitative differences, which distinguish *P. indica* on *H. vulgare* (and *A. thaliana*) from endomycorrhizal fungi. The fungal root colonization increases with root maturation and the highest fungal biomass has been found in the differentiation, particularly in the root hair zones. Cytological studies have revealed the diverse types of *P. indica*-interaction with different root regions of *H. vulgare*, where the root hair zone (as the oldest root zone) was observed to be highly colonized by intracellular hyphae (Deshmukh et al., 2006). Cells in the differentiation zone can be filled with fungal hyphae reminiscent of hyphal coils (Deshmukh et al., 2006); whereas, scarcely and solely extracellularly colonization can be evidenced in the cells of meristematic zone (Schäfer and Kogel, 2009). Importantly, the physiological activity of host cells has been considered as a prerequisite for efficient nutrient exchange between the symbiotic partners (Schäfer and Kogel, 2009). Thus, root colonization pattern of *P. indica* differs from that of AM fungi, which are known to preferentially colonize younger root parts (Schäfer and Kogel, 2009) (**Figure 1**).

Zuccaro et al. (2011) presented the first in-depth genomic study and unveiled a mutualistic symbiont with a biphasic lifestyle. On the 25 Mb genome of the mutualistic root symbiont *P. indica*, the authors characterized fungal transcriptional responses associated with the colonization of living and dead *H. vulgare* roots. A biphasic root colonization strategy of *P. indica* was revealed by microarray analysis, where a tightly controlled expression of the lifestyle-associated gene-sets was reported during the onset of the symbiosis. It has been observed that about 10% of the fungal genes induced during the biotrophic colonization encoded putative small secreted proteins (SSP). SSP included several lectin-like proteins and members of a *P. indica*-specific gene family (DELD) with a conserved novel seven-amino acids motif at the C-terminus (Zuccaro et al., 2011). The occurrence of the DELDs was further correlated with the presence of transposable elements in gene-poor repeat-rich regions of the genome similar to the effectors found in other filamentous organisms. These findings together, helped to understand the development of biotrophic plant symbionts and also suggested a series of incremental shifts along the continuum from saprotrophy toward biotrophy in the evolution of mycorrhizal association from decomposer fungi (Zuccaro et al., 2011).

On the perspective of the biotrophic colonization pattern of *P. indica*, it has been reported that the *P. indica* biotrophic colonization pattern can be accompanied by a broad-spectrum suppression of root innate immunity (Qiang et al., 2011). In the support of the large host range of *P. indica*, molecular and genetic analyses revealed that plant roots, similar to leaves, are equipped with an effective innate immune system where immune suppression by *P. indica* was considered as a prerequisite for successful root colonization (Jacobs et al., 2011; Qiang et al., 2011). A little work has been performed in order to decipher the underlying mechanisms of suppression of root innate immunity by *P. indica* for a successful colonization. However, there are evidences that signify the involvement of several phytohormones in this context (Qiang et al., 2011). *H. vulgare* and *Arabidopsis* mutants impaired in gibberelic acid (GA) and jasmonic acid (JA) metabolism, respectively exhibited elevated root immune responses together with reduced root colonization (Schäfer et al., 2009; Jacobs et al., 2011). Studies have also revealed the dependency of *P. indica* on JA-mediated suppression of early immune responses (e.g., root oxidative burst) as well as salicylic acid (SA)- and glucosinolate-related defense pathways (Jacobs et al., 2011). For example, *Arabidopsis* mutants impaired in SA- and glucosinolate-associated defense were reported to be more susceptible to *P. indica* (Sherameti et al., 2008; Jacobs et al., 2011). The disturbance of endoplasmic reticulum (ER) integrity by *P. indica* has been advocated to have potential for impairing the secretion of immunity-associated proteins (e.g., PR1 and PRRs) (Qiang et al., 2011). This suppression of early immune signaling (which disturbs immune execution) may potentially disarm the root, and thereby may facilitate root colonization. Since the genome of *P. indica* is available, it is possible to identify effector molecules targetting immune signaling components (Qiang et al., 2011).

## *Piriformospora indica*–Plant Mutualistic Interaction and the Role of Calcium Ions

Both mutualists and commensals are identical for many fungi during the initial phases of infection and colonization by pathogens (Rodriguez et al., 2004). Thus, the mode of recognition and early signaling processes are crucial in understanding how plants can differentiate between a beneficial and a detrimental microbe which in turn can modulate the expression of lifestyle in plants (Vadassery and Oelmüller, 2009; Singh et al., 2011). Notably, within seconds or minutes after the recognition of the two partners, an increase in the level of intracellular calcium (Ca^2+^) in a plant cell has been considered as an early signaling event in the interaction of pathogenic, mycorrhizal or endophytic microbes with plants (McAinsh and Pittman, 2009). Nevertheless, in various plant-signaling pathways, Ca^2+^ ions act as a second messenger in order to couple extracellular stimuli with intracellular and whole plant responses (Sanders et al., 2002). Endophytic and mycorrhizal fungus interactions result in a better plant performance through sequential cytoplasmic and nuclear Ca^2+^elevations (Vadassery and Oelmüller, 2009).

Ca^2+^ ions have been evidenced as a key participant in the mutualistic interaction of both *P. indica* and *Arabidopsis*. It is also one of the earliest signaling events during the recognition of these two symbionts, where a rapid induction of [Ca^2+^]_cyt_ elevation follows a nuclear Ca^2+^ response (Vadassery et al., 2009). Quite a few mutants which do not respond to *P. indica* concerning growth promotion and higher biomass production are also impaired in [Ca^2+^]_cyt_ elevation. Additionally, elevations in the [Ca^2+^]_cyt_ can also be induced by an autoclaved cell wall extract (CWE) from *P. indica*, which also can promote growth of *Arabidopsis* and other plant species (Vadassery et al., 2009). Previous facts together suggest insignificance of root colonization by the living fungus in the highlighted above response. Inductions in the elevation of [Ca^2+^]_cyt_ by autoclaved CWE, preferentially in the roots confirm that the endophyte is a root-colonizing fungus. The very same CWE can induce a slightly different Ca^2+^ signature in tobacco roots hinting at the possibility of species-specific plant responses. CWE from *P. indica* also induces tuberization *in vitro* and promotes tuber growth and yield in potato due to increased transcript expression of the two Ca^2+^ dependant proteins (such as CaM1 and St-CDPK1) and the lipoxygenase (LOX) mRNA, which are known to play distinct roles in potato tuberization (Upadhyaya et al., 2013).

**FIGURE 2:**
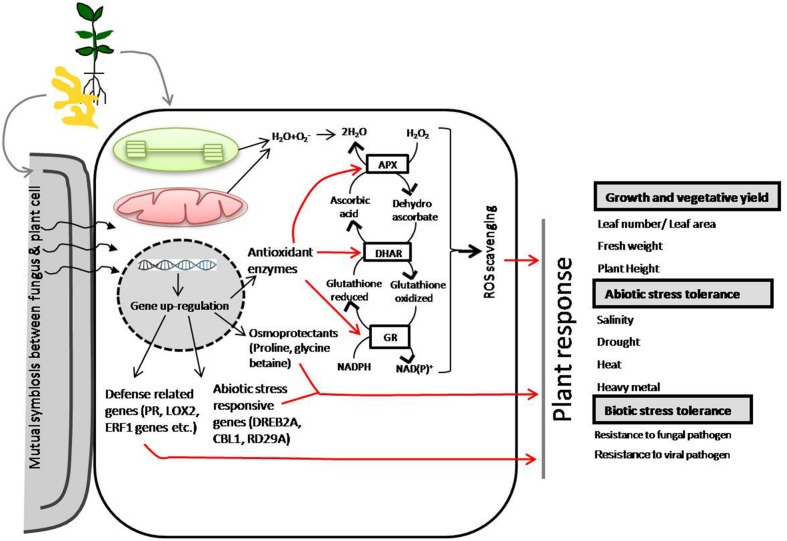
**Schematic representation of *Piriformospora indica* symbiotic association-mediated crop/plant abiotic and biotic stress tolerance (Camehl and Oelmüller, 2010; Sun et al., 2010; White and Torres, 2010; Foyer and Shigeoka, 2011; Molitor et al., 2011; Hamilton et al., 2012; Zarea et al., 2012)**.

At the contact surface of plants, *P. indica* exchanges various signals which can result into influx of phosphorus and eﬄux of Ca^2+^ within plant cell (Yadav et al., 2010; Ansari et al., 2013). The external stimuli from endophyte signaling cascade of cellular network either signifies phytohormones to mainly be involved in PCD *via* endoplasmic reticulum stress or directly be involved in growth and development (Qiang et al., 2011; Ansari et al., 2013). Exhibition of the reduced colonization, plant growth and development were reported by the plant mutants impaired in GA and JA metabolism (Schäfer et al., 2009; Jacobs et al., 2011). Generally, in PCD, Ca^2+^ and phytohormone signal as internal or external stimuli that down-regulates (*PR1b*, *PR5*, *β-gulcanase* etc.) and up-regulates (auxin) (*BI-1* gene) (Hückelhoven, 2004; Lam, 2004; Deshmukh and Kogel, 2007; Trivedi et al., 2013). PCD as DNA fragmentation and cell shrinkage were common during *P. indica* colonization (Deshmukh et al., 2006). The host cell death in *H. vulgare* plants *via* constitutively overexpressing the negative cell death regulator Bax Inhibitor-1 (*BI-1*) was reported to reduce colonization of roots (Deshmukh et al., 2006). *Arabidopsis* roots displayed ultrastructural alterations at the time of cell death that was linked with the colonization by *P. indica*. This can be explained by the fact that *P. indica* induces ER stress in colonized roots and also suppresses the adaptive ER stress response pathway (unfolded protein response, UPR) (Qiang et al., 2011, 2012). The inability of colonized cells to relieve ER stress *via* the UPR leads to activation of a pro-apoptotic signaling cascade (Qiang et al., 2011, 2012). Phytohormones and Ca^2+^ (which might occur at the plant-fungus interface) can direct the molecular and physiological processes responsible for the actual mechanism of *P. indica* colonization with plants providing various benefits to plants (**Figure 2**).

## *Piriformospora indica* and Programmed Cell Death

Generally, PCD is a natural response of plants to face physiological constraints provoked by varied internal or external stimuli (Hückelhoven, 2004; Lam, 2004). PCD is a metabolically regulated mechanism (e.g., nutrient recycling) vital for plant development. However, in the case of hypersensitive response (HR), PCD has a protective function in local and systemic tissue, characterized by defense gene expression to check the growth and propagation of pathogens (Heath, 2000; Hoeberichts and Woltering, 2003). Plant-PCD shares some common characteristics to apoptosis in animals such as chromatin condensation, cell shrinkage and DNA fragmentation followed by the nucleus breakdown (Heath, 1998). The fungal colonization of roots begins with a biotrophic growth phase, in which living cells are colonized, and followed by a cell death-dependent phase, in which root cells are actively killed by the fungus (Qiang et al., 2011). Earlier, *P. indica* was reported to colonize roots (such as that of *Arabidopsis*) by an initial biotrophic phase, which was evidenced to follow a cell death-dependent phase that may not show any disease symptoms on plant roots (Deshmukh et al., 2006). DNA fragmentation and cell shrinkage, common features of PCD can also be observed in *P. indica*-colonized roots (Deshmukh et al., 2006). It is assumed that *P. indica* uses these dead cells for intracellular sporulation (Qiang et al., 2011). In contrary to their pronounced induction during pathogen-induced HR (Heath, 2000), Deshmukh and Kogel (2007) argued that the kind of cell death occurred during *P. indica* colonization in *H. vulgare* may not be regarded as a microorganism-antagonizing plant response, since the defense marker genes (e.g., *PR1b*, *PR5* and *1,3-β-glucanase*) are weakly and transiently up-regulated solely at early interaction stages. Besides, accumulation of ROS (e.g., hydrogen peroxide, H_2_O_2_), as well as whole-cell auto-fluorescence mediated by phenolic compounds as characteristics of HR (Lamb and Dixon, 1997; Heath, 2000; Apel and Hirt, 2004) have never been detected in the *P. indica* colonized roots (Schäfer et al., 2007).

Transmission electron microscopic studies have revealed that cells are not dead at the penetration stages but show ultrastructural changes as cell-colonization is ascertained (Qiang et al., 2011). It implies that the fungal-colonization strategy is not merely focused on the perception and subsequent colonization of dead cells though the penetrated host cells certainly die at one defined point of cell-colonization (Schäfer and Kogel, 2009). Evidences also confirm the dependency of this colonization strategy merely on the host cell death. Reduced colonization of roots was observed in *H. vulgare* plants constitutively overexpressing the negative cell death regulator Bax Inhibitor-1 (*BI-1*) (Deshmukh et al., 2006). The expression of *BI-1* in *P. indica*-infested *H. vulgare* was suppressed from 5 days after inoculation onward, which corresponded with cellular mycelial proliferation, and did not match with earlier root penetration events. Additionally, in comparison to the wild-type plants, the constitutive overexpression of *HvBI-1* in *H. vulgare* led to a significant reduction in fungal biomass at 20 days after inoculation. These findings also agreed with the significant role of *BI-1* in plant defense and cell survival (Schäfer et al., 2007). *BI-1*, known to be an integrator of ER stress, supports cell integrity and viability under unfavorable conditions. Besides its involvement in cell death, the ER is also significant for correct processing of immunity-related proteins with an extracellular (Wang et al., 2005) or plasma membrane-associated destination, including the pattern recognition receptor EFR (Nekrasov et al., 2009; Saijo et al., 2009). After recognition of the bacterial elongation factor TU, EFR activates basal immunity and effectively halts bacterial invasion of plants (Zipfel et al., 2006). Recent cellular analyses of *Arabidopsis* roots revealed ultrastructural alterations during cell death-associated colonization by *P. indica* (Qiang et al., 2011, 2012). According to these studies, *P. indica* induces ER stress in colonized roots, nevertheless, at the same time suppresses the adaptive ER stress i.e., unfolded protein response pathway (UPR). The inability of colonized cells to relieve ER stress *via* the UPR leads to activation of a pro-apoptotic signaling cascade. Thus, vacuolar collapse was identified to be downstream of ER stress and to represent a key element of the *P. indica*-induced cell death pathway. This vacuolar collapse has been considered essential for both cell death execution and root colonization, which is mediated by vacuolar processing enzymes (VPEs). For example, *Arabidopsis* mutants lacking VPEs were found incapable of undergoing cell death-associated vacuolar collapse and exhibited reduced fungal colonization (Qiang et al., 2011, 2012). It has been suggested that *P. indica* achieved its large host range through a biphasic colonization strategy, which consists of biotrophic accommodation by effective host immune suppression, followed by an ER stress-induced caspase-dependent vacuolar cell death. The exact execution of both colonization phases is a prerequisite for a successful symbiosis with *Arabidopsis* (Qiang et al., 2011, 2012). *PYK10*, a gene for an abundant β-glucosidease/myrosinase of 65 kDa is located in the ER (Nitz et al., 2001; Matsushima et al., 2004) and has been identified as a target of *P. indica* in *Arabidopsis* roots (Peškan-Berghöfer et al., 2004; Sherameti et al., 2008). *PYK10* was argued as a requirement for the beneficial interaction between *Arabidopsis* and *P. indica* (Sherameti et al., 2008). In fact, non-toxic glucosinolates can be hydrolyzed by myrosinases to biologically active isothiocyanates, thiocyanates, nitriles or epithio nitriles (Rask et al., 2000; Wittstock and Halkier, 2002). However, the nature of the aglycon moieties released from the substrates basically controls the biological function of a myrosinase. The involvement of these aglycons in the plant defense against herbivores and microbes has been reported (Stotz et al., 1999, 2000; Rask et al., 2000; Tierens et al., 2001). Thus, myrosinases can be exploited for their applications in plant biotic/abiotic stress defense.

Sherameti et al. (2008) hypothesized that the broad host range of *P. indica* is possibly due to its interaction(s) based on general recognition and signaling processes. In order to identify plant genes, which are targeted by the fungus, *Arabidopsis* mutants were screened that do not respond to the fungus with regard to growth promotion and enhanced seed production (Oelmüller et al., 2004; Shahollari et al., 2007). The authors reported that the growth of a T-DNA insertion line in *PYK10* is not promoted and the plants do not produce more seeds in the presence of *P. indica*, although their roots are more colonized by the fungus in comparison with the wild-type roots. Overexpression of *PYK10* mRNA did not affect either root colonization or the response to the fungus. The basic helix-loop-helix domain containing transcription factor *NAI1* activates expression of *PYK10*, and two *Arabidopsis* lines with mutations in the *NAI1* gene show similar response to *P. indica* as that of *PYK10* insertion line. *PYK10* transcript and *PYK10* protein levels are severely reduced in a *NAI1* mutant, signifying that *PYK10* is responsible for the response to the fungus not the transcription factor *NAI1*. The message level for a leucine-rich repeat protein *LRR1* is upregulated in wild-type roots in the presence of *P. indica* but not for plant defensin 1.2 (PDF1.2). Contrary to that, the message level for PDF1.2 is upregulated in the presence of the fungus in lines with reduced PYK10, not for LRR1. Sherameti et al. (2008) concluded that PYK10 restricts root colonization by *P. indica*, which leads into repression of defense responses and the upregulation of responses directing to a mutualistic interaction between the two symbiotic partners.

## *Piriformospora indica* Genome – Insights to Surprise

A comparison of the *P. indica* genome with other fungi has revealed its classical features related with biotrophism as well as saprotrophism. The colonization of *P. indica* with *Arabidopsis* roots has been report to involve an initial biotrophic phase followed by cell death dependent phase, leading ultimately to no disease symptoms on roots (Deshmukh et al., 2006). However, *P. indica* was reported to use the dead cells formed therein during the course of infection for intracellular sporulation (Qiang et al., 2011). The endophyte infection was earlier considered entirely symptomless, involving certain genes to indicate systemic root colonization by different fungal groups (Waller et al., 2008). Exhibition of biotroph-associated genomic adaptations has been evidenced in *P. indica*, where genes involved in N metabolism are lacking and also a limited potential is displayed by this mutualistic symbiont for host-damage and destruction (Zuccaro et al., 2011). In fact, *P. indica* lacks genes potentially involved in biosynthesis of toxic secondary metabolites and cyclic peptides. Nevertheless, genomic traits with saprotrophic and hemibiotrophic phytopathogenic fungi (such as the presence of an expanded enzyme arsenal which is weakly expressed during the initial biotrophic phase) are also shared by *P. indica* (Zuccaro et al., 2011). The analyses of the colonized young *H. vulgare* roots has revealed 579 genes in the phase of pre-penetration (*36–48 hpi*), 397 genes at early colonization (*3 dpi*) and 641 genes (*5 dpi*) as distinctively regulated when compared to fungal free roots (Zuccaro et al., 2011). In addition, the majority of genes (≈40%) induced *via* symbiosis were found to be non-orthologous to either species; rather, these were reported specific to *P. indica* (Zuccaro et al., 2011). Plant hormones such as JA, methyl-jasmonate and ethylene signals from the roots were reported to decide the shoots to become preconditioned prior to foliar pathogens infection in *P. indica*-colonized roots *via* activating defense responses which in turn were evidenced to lead to enhanced disease resistance (Stein et al., 2008). In a signaling cascade, the JA (*VSP*, *PDF1.2*, *LOX2*) plus ET (*ERF1*) signaling and not the SA signaling (*PR1*, *PR5*) genes were up-regulated in the *P. indica* plants in order to cope the powdery mildew fungus (Waller et al., 2005; Unnikumar et al., 2013). The indole-3-acetic acid (IAA) and ethylene role has been implicated in establishing a biotrophic symbiosis representing a compatibility factor at contact surface between endophyte and plants (Hilbert et al., 2012; Khatabi et al., 2012). The augmented intracellular Ca^2+^ pool after attaining the basic compatibility between the two partners in an early signaling event in the interaction of endophyte with plants (McAinsh and Pittman, 2009), acts as a second messenger in various plant signaling pathways (Sanders et al., 2002).

## *Piriformospora indica*-Services to Plant Community

### Nutrient Acquisition

Soil signifies a positive environment for a wide range of microorganisms including algae, bacteria, and fungi and the chemical changes that happen within the soil environment involve the active contribution of soil microflora (Prasad et al., 2015a). They chiefly participate in the processes which are necessary for plant growth and survival such as C and N cycle, nutrient acquisition and soil formation. On the other hand, owing to their role in C-input in soils through root exudates, plants can also have profound effects on soil microbial communities especially those colonizing the rhizosphere (Khatabi, 2009).

Being immobile organisms, plants have to cope with unfavorable conditions such as nutrient deficiency, salinity, drought, and pathogen attacks etc. Thus, to avoid such adverse situations, plants tend to establish their associations with beneficial microorganisms (Lum and Hirsch, 2003). In particular, symbiosis with beneficial fungi are known to be vital for nutrient acquisition by the root systems of most plants (Sirrenberg et al., 2007). Thus, application of beneficial microorganisms as biofertilizer plays a key role in today’s agricultural scenario through enhancement of soil fertility and crop production. For example, mycorrhizal fungi and AM fungi under the order Glomales, are known to have symbiotic relationship with the majority of terrestrial plant species, where the fungi facilitate plants in their accession to soil nutrients, mainly phosphate (Harrison, 2005). Interestingly, AM symbiosis is described relatively primordial with that of rhizobium-legume association where ‘Myc factor,’ a AM fungal signal, is a mixture of sulphated and non-sulphated simple lipochitooligosaccharides, might turn on signaling pathways having similar components of doesn’t make infections (DMI)-signal transduction pathway to establish the mycorrhiza formation to promote plant growth (Maillet et al., 2011). Endophytic fungi *P. indica* which is phylogenetically close to mycorrhizal endosymbionts of orchid and ericoid roots has also been recognized as a growth promoter of numerous plant species (Varma et al., 1999; Weiss et al., 2004). Although mutual nutrient exchange through specialized structures is generally accepted as the main beneficial factor in mycorrhiza, however, major mechanisms of interaction in other endophytic systems are still not clear.

### *P. indica* and Acquisition of Phosphorous in Plants

Phosphorous (P), one of the most essential mineral nutrients constitutes up to 0.5% of the dry weight of plant cell, and plays diverse regulatory, structural, and energy transfer roles (Balemi and Negisho, 2012). Plants cannot directly access P present in the soil as it is mostly in the form of scarcely soluble complexes; hence, its deficiency impedes crop production throughout the world (Balemi and Negisho, 2012). Plants acquire P from the soil through direct uptake by its own transporters and indirect uptake through mycorrhizal associations (Yadav et al., 2010). *P*. *indica* was reported to mediate the uptake of radiolabelled P from the culture medium and its translocation to the host in an energy-dependent process (Varma et al., 2000). *P*. *indica* also produce significant amounts of acid phosphatases which can enable the host plant to access adequate amount of insoluble, condensed or complex forms of phosphate reserve in the soil (Singh et al., 2000). The association of P ectomycorrhiza and its role in plant P-aacquisition has been the focuss of recent studies (Becquer et al., 2014; Johri et al., 2015). Identification and characterization of high affinity phosphate transporters were also done in several plant and fungal species including *A. thaliana*, *Medicago truncatula*, *Lycopersicon esculentum*, *Solanum tuberosum*, *Saccharomyces cerevisiae*, and *Neurospora crassa* (reviewed by Johri et al., 2015). Notably, due to the lack of a stable transformation system in AM fungal species the role of phosphate transporters could not be established (Maldonado-Mendoza et al., 2001). Isolation, identification, and functional characterization of a high affinity phosphate transporter from root endophyte fungus *P. indica* revealed the essentiality of phosphate transporter (PiPT) for phosphate transport to the host plant (Yadav et al., 2010). Nevertheless, the mechanism of this phosphate transfer from the fungus to the plant is not yet clear, though it has been hypothesized that the process might occur at the plant-fungus interface. This process essentially requires two transporters: the first to enable eﬄux of phosphate from the fungus and the second to mediate uptake of phosphate by the plant (Rausch and Bucher, 2002).

The reports concerning the involvement of *P. indica* in phosphate transfer and improvement in host plant are contradictory. Shahollari et al. (2005) reported that *P. indica* enhances the phosphate uptake 2–3 times higher in *Arabidopsis* seedlings and suggested that *P. indica* stimulates *Arabidopsis* growth in a manner parallel of mycorrhizal fungi. On the contrary, it has also been reported that *P. indica* does not induce significant increase of leaf P and N and phosphate has no role in the improved biomass of *Nicotiana attenuata* (Barazani et al., 2005). Yadav et al. (2010) reported that *P. indica* is involved in the phosphate transfer to the host *Zea mays* plant and proposed that involvement of *PiPT* in indirect phosphate transport to host plants presents the information regarding the molecular mechanism underlying *P. indica*-mediated phosphate transport to the host plant. However, it was resported earlier that *P. indica* does not induce potato phosphate transporter gene (*StPT3*) and that *P. indica* is not involved in the phosphate transfer to host plant (Karandashov et al., 2004). Correspondingly, no improved phosphate supply (a central mechanism of host plant fortification by AM fungi) was evidenced in *P. indica*–*H. vulgare* symbiosis (Achatz et al., 2010). However, the improved grain yield induced by the fungus was found to be independent of different P and N fertilization levels. Also, the total phosphate contents of host plant roots and shoots were not significantly affected by *P. indica*. The authors concluded that positive influence of *P. indica* on *H. vulgare* grain yield was independent of P and N supply and rather due to accelerated growth that leads to early development (Achatz et al., 2010).

Contrary to the reports discussed above, Yadav et al. (2010) found the impact of phosphate on the biomass of the *Z. mays* plant colonized with *P. indica*. Increased total phosphate content as well as biomass in the plants colonized with wild-type *P. indica* as compared with non-colonized and KD*-PiPT P. indica*-colonized plants were observed. These findings imply that phosphate play a significant role in the improvement of yield or biomass of *Zea mays*, and that enhanced biomass is in fact due to the *PiPT*. Furthermore, the growth-promoting activity (in terms of biomass) of *P. indica* was two-fold higher at low phosphate condition as compared with high phosphate condition (1.2-fold). Kumar et al. (2011) further investigated to ascertain whether growth-promoting activity of *P. indica* is an inherent trait of the fungus or it is dependent on the availability of P, under P-rich and -deprived conditions. No significant change in the biomass was found at seedling stage; however, the difference in biomass was significant between colonized plants grown in P deprived conditions and non-colonized plants in rich P conditions at later stage. *P. indica* enhances biomass more efficiently in P-deprived condition than P-rich condition. The growth of colonized plant at 40 μM phosphate was equal to non-colonized plants grown at 400 μM indicating that *P. indica* can mimic the growth even at 10-fold lower P supplement. Bicompartment assay for ^32^P transportation revealed that the fungal hyphae transport P to host plant in P-deprived condition but unable to transfer more P in P-rich condition though, there was no difference in the colonization at both the conditions. Hence, it has been concluded that *P. indica* has an ability to increase the biomass of the maize plant specifically under low phosphate condition thus; *P. indica* could be a good candidate for utilization in sustainable agriculture for the improvement of crop production in land deficient in P (Yadav et al., 2010; Kumar et al., 2011). Furthermore, RT-PCR analysis showed the expression of *PiPT* gene in P-deprived condition but not in P-rich condition. Therefore it was concluded that *PiPT* is actively involved in transporting P to the seedlings and that *PiPT* expression is dependent on the P availability. Though the mechanism for the transfer of P from *P. indica* to plant is not fully understood; however, it was hypothesized that Pi and organic P (such as polyphosphate) can be carried within the fungus by cytoplasmic streaming or by bulk flow to the plant root from external hyphae located in the soil (Kumar et al., 2011).

Conflicting results discussed above may be due to the host-specific nature of *P. indica* since all the studies were conducted on different crop plants. Therefore, a complete range of different host plants would only provide a clear picture of whether *P. indica* and *PiPT* are host-specific or not. Exploitation of *P. indica* and its *PiPT* not only can complement crop improvement strategies but may also serve as a model system to study molecular mechanism and indirect uptake of phosphate by plants (Yadav et al., 2010). Recently, the crystal structure of PiPT has been elucidated (Pedersen et al., 2013). With reference to the root colonization strategy of *P. indica*, i.e., the program cell death (Deshmukh et al., 2006), the authors emphasized that the main part of the root further develops and is not necrotized when colonized by the fungus. Hence, it was hypothesized that once the fungus releases phosphate into dead cells, it might be taken up by the non-affected living adjacent cells and further distribute into different parts of the plant (Deshmukh et al., 2006). Future research should be designed to perform gene function analysis studies in *P. indica* which has been a major constraint in genetic manipulation this fungus until now. In this context, the recently established electroporation-mediated transformation system based on the polyethylene glycol method for *P. indica* (Zuccaro et al., 2009) in combination with RNAi-mediated gene silencing (De Backer et al., 2002) have been suggested as major genetic engineering tools (Yadav et al., 2010).

### *P. indica* and Acquisition of other Major Nutrients in Plants

Deficiency of other important nutrients such as nitrogen (N) (Xu et al., 2012) and zinc (Zn) (Tsonev and Lidon, 2012) in soil has been reported to restrict plant growth and development. Plants recruit N either as nitrate or ammonium but in some species by N fixation with the help of rhizobia (Esseling and Emons, 2004). Mycorrhizal fungi also play an important role in delivering either nitrate or ammonium to the root cells. It is believed that mycorrhizal fungi preferentially recruit ammonium rather than nitrate from the soil and that amino acids represent the major compounds that serve to transfer nitrogen to the host plant (Guescini et al., 2003). Sherameti et al. (2005) reported that the co-cultivation of *Nicotiana tabaccum* and *Arabidopsis* seedlings with *P. indica* is accompanied by a huge transfer of N from the agar plates into the aerial part of the seedlings. This effect is associated with activation of the NADH-dependent nitrate reductase (NR), the enzyme which plays a key role in nitrate acquisition in plants. However, the stimulation of nitrate assimilation by *P. indica* is the reason for the growth promotion is not known. *P. indica* activates NR which in turn plays a key role in nitrate acquisition and also a starch-degrading enzyme, glucan-water dikinase is involved in the early events of starch degradation in *N*. *tabaccum* and *Arabidopsis*. *P. indica* also activates the expression of the genes for the starch-degrading enzyme, glucan-water dikinase (*SEX1*) in roots which is involved in early events of starch degradation in *N. tabaccum* and *Arabidopsis*. Both the growth promotion and stimulation of the two enzymes do not require heterotrimeric G-proteins. *P. indica* also stimulates the expression of the *uidA* gene under the control of the *Arabidopsis* NR (*Nia2*) promoter in transgenic tobacco seedlings. A homeodomain transcription factor responds to the fungus and binds to promoter regions of the *P. indica*-responsive *Nia2*, *SEX1*, and 2-nitropropane dioxygenase genes, indicating that the expression of *P. indica*-responsive target genes may be controlled by common regulatory elements and *trans*-factors. It is proposed that the growth promoting effect initiated by *P. indica* is accompanied by a co-regulated stimulation of enzymes involved in nitrate and starch metabolisms. Application of bioinoculants prepared with the of fluorescent pseudomonas strains R81, and *P. indica* can efficiently promote growth in tomato plants probably due to the colonization strategies of plant growth promoting rhizobacteria (PGPR) and *P. indica* (Sarma et al., 2011). It has also been suggested that the better root density of the inoculated plants facilitate and improve uptake of the nutrients from the rhizosphere. Fluorescent pseudomonad strain (R81) being a phosphate solubilizing strain (Roesti et al., 2006) and acid phosphatases produced by *P. indica* were reported to mobilize and assist *L. esculentum* plant in phosphate-acquisition, and growth promotion (Sarma et al., 2011). Recently, a model has been proposed to study an interaction between different strains of *Azotobacter* and *P. indica* (Bhuyan et al., 2015). The interaction with *P. indica* is proposed as a useful tool for mitigating zinc (Zn)-deficiency stress in *Triticum aestivum* (Abadi and Sepehri, 2015). Improved uptake of mineral nutrients, antioxidant enzyme activities, photosynthetic pigments, and low lipid peroxidation have been observed in *T. aestivum* plants under Zn-deficiency conditions with co-inoculation of two plant-growth-promoting microorganisms; *Azotobacter chroococcum* and *P. indica* (Abadi and Sepehri, 2015).

### Seed Germination, Plant Growth and Development and Productivity

*Piriformospora indica* can also significantly mediate improvements in the growth and yield of various crop plants, horticultural and medicinal plants (Varma et al., 2001; Peškan-Berghöfer et al., 2004; Pham et al., 2004; Vadassery et al., 2008; Kumar et al., 2009; Oelmüller et al., 2009; Achatz et al., 2010; Fakhro et al., 2010; Gosal et al., 2010; Sun et al., 2010). *P. indica*-induced seed germination and development have been reported in several crop plants (Varma et al., 2012a,b, 2013). *P. indica*-mediated seed development and enhanced seed production in *A. thaliana* were reported as a result of the *pii-2* and At5g16590 located in the micro-domains of plasma membrane (Shahollari et al., 2007). *P. indica*-inoculated *H. vulgare* seeds exhibited higher viability (Harrach et al., 2013). Moreover, germinated seedlings immersed in *P. indica*-homogenate exhibited a good survival rate under adverse conditions (Harrach et al., 2013). *P. indica* filtrate can facilitate early seed germination in vascular plants (Adya et al., 2013). In *Helianthus annus*, *P. indica* culture filtrate was evidenced to influence the seed-oil yield (Bagde et al., 2011). A higher seed yield was also reported in the members of bryophytes, pteridophytes, gymnosperms and angiosperms as a result of the colonization of *P. indica* in their roots (Varma et al., 2012a,b) (**Table 1**).

**Table 1:** Summary of representative recent studies highlighting beneficial roles of *Piriformospora indica* in major crops/plants under normal, and biotic and abiotic stress conditions.

Plants/host plants	Beneficial roles	Reference
**Normal/non-stress conditions**		
*Hordeum vulgare*	Increase in seed viability and survival, and vegetative and grain yields	Harrach et al., 2013
*Brassica campestris* sp. *Chinensis*	Increase in root and shoot fresh weight	Sun et al., 2010; Lee et al., 2011
*Tridax procumbens*	Increase in root and shoot length and fresh and dry weight	Das et al., 2013
*Cyclamen persicum*	Increase in the numbers of flowers and unfolded leaves; Increase in the proportion of homogeneous microspores and viable pollen and ovules	Ghanem et al., 2014
*Foeniculum vulgare*	Increase in plant height, shoot and root dry weight, number of inflorescence	Dolatabadi et al., 2012
*Helianthus annus*	Higher seed yield with increased oil content, Lipid biosynthesis	Bagde et al., 2011
*Centella asiatica*	Increase in plant fresh weight, leaf and root number	Satheesan et al., 2012
*Jatropha* and *Populus*	Early seed germination, and increase in seed formation and seed yield	Varma et al., 2013
*Oryza sativa*	Improved root and shoot length and dry weight	Jogawat et al., 2013
*Nicotiana attenuata*	Increase in stalk length, number of flower/plant, seed weight and root fresh weight	Schuck et al., 2012
*Lycopersicon esculentum* (Tomato)	Increase in seedling growth	Anith et al., 2015
Vegetable crops	Induced seed germination, Seed formation, seed value and yield	Varma et al., 2012a,b, 2013
**Biotic stresses**		
*A. thaliana*	Significant reduction in *Verticillium dahlia*-mediated disease development	Sun et al., 2014
*A. thaliana*	Protection against verticillium wilt and root rot caused by *Verticillium longisporum* and *Rhizoctonia solani*, respectively.	Knecht et al., 2010
*A. thaliana*	Protection against leaf blight caused by *Alternaria brassicae*	Johnson et al., 2013
*A. thaliana*	Protection against Verticillium wilt caused by *V. dahlia*	Sun et al., 2014
*H. vulgare*	Protection against rhizoctonia root rot caused by *Rhizoctonia solani*	Qiang et al., 2012
*L. esculentum*	Protection against Fusarium wilt and black root rot caused by *Fusarium oxysporum* and *Thielaviopsis basicola*, respectively.	Qiang et al., 2012
*L. esculentum*	Protection against yellow leaf mosaic and Verticillium wilt caused by Pepino mosaic virus and *V. dahlia*, respectively	Fakhro et al., 2010
*Triticum aestivum*	Protection against *Fusarium* head blight disease isolates and mycotoxin (deoxynivalenol) contamination	Rabiey et al., 2015
**Abiotic stresses**		
*Hordeum vulgare*	Increase salinity tolerance as indicated by increasing the foliar potassium (K^+^)/sodium (Na^+^) ratio	Alikhani et al., 2013
*H. vulgare*	Drought stress tolerance	Ghabooli et al., 2013
*H. vulgare*	Increases in the biomass of aerial parts; increased the K^+^/Na^+^ and Ca_2_^+^/Na^+^ ratios, and increase in salinity tolerance	Ghabooli, 2014
*H. vulgare*	Increase in crop yield under low temperature stress	Murphy et al., 2014
*Nicotiana tabacum*	Enhanced cadmium tolerance	Hui et al., 2015
*Oryza sativa*	Increase in salinity stress	Jogawat et al., 2013
*Sesamum indicum*	Increase in growth and tolerance to drought stress	Zhang et al., 2014
*T. aestivum*	Increase in cadmium stress tolerance	Shahabivand et al., 2012
*Solanum lycopersicum*	Osmotic stress and chloride toxicity	Al-Absi and Al-Ameiri, 2015
*T. aestivum*	Mitigation of zinc deficiency stress	Abadi and Sepehri, 2015

The role of *P. indica* inoculation/colonization in medicinal plants has been considered of utmost significance (Das et al., 2012). The colonization of *P. indica* has been reported in a number of medicinal plants including *Coleus forskohlii, Bacopa monnieri, Stevia rebaudiana, Artemisia annua, Linum album, Trigonella foenumgraecum, Spilanthes calva, Withania somniferra, Chlorophytum borivilianum, Curcuma longa, Podophyllum peltatum, Azadirachta indica, Oscimum sanctum*, *Linum album* and *Lantana camara* (Oelmüller et al., 2009; Das et al., 2012; Sharma and Agarwal, 2013; Kumar et al., 2013, 2015; Ahlawat et al., 2015) (**Table 2**). Increased contents of chemical compounds and secondary metabolites were reported in *P. indica*-colonized *C. forskohlii* (Das et al., 2012). *P. indica* has also been reported to enhance the growth, bacoside endogenous level, antioxidant activity as well as hypertrophy of nuclei in *B. monnieri* (Prasad et al., 2013). Medicinal properties of *A. indica, A. elegans*, and *H. annuus* were increased with their colonization with *P. indica* (Bagde et al., 2010b). *P. indica*-inoculation in *L. album* cell culture improved the production of anticancer drug podophyllotoxin (Kumar et al., 2013). Leaf area and fresh biomass were improved in herbaceous plants namely *S. rebaudiana* and *A. annua* with their cultivation with *P. indica* (Varma et al., 2013). Earlier, Rai et al. (2001) reported a positive response of the growth of medicinal plants such as *S. calva* and *W. somnifera* in a field trial with *P. indica* inoculum (**Table 2**). Recently, *in vitro* co-cultivation of *P. indica* filtrate was reported to improve biomass productivity in *A. annua* and *W. somniferra* (Ahlawat et al., 2015; Baishya et al., 2015). Cell suspension cultures of *W. somnifera* offers the potential for continuous production of withaferin A (Ahlawat et al., 2015). Further, it can be concluded that the symbiotic effect of *P. indica* can be used as a biopriming agent for the overall growth of plant biomass.

**Table 2:** Summary of representative recent studies highlighting beneficial roles of *Piriformospora indica* in major medicinal plants.

Plants/Host plants	Beneficial roles	Reference
*Aloe vera*	Improved micropropagation, growth and phytochemical content	Sharma et al., 2014
*Artemisia annua*	Increased biomass productivity	Baishya et al., 2015
*Azadirachta indica, Aristolochia elegans*, and *Helianthus annuus*	Enhanced biomass production and increased medicinal property and yield	Bagde et al., 2010a
*Bacopa monnieri*	Increase in growth, and bacoside endogenous level and antioxidant activity	Prasad et al., 2013
*Coleus forskohlii*	Increase in growth parameters, aerial biomass and in important metabolites production for medicinal application	Das et al., 2012, 2014
*Curcuma longa*	Increase in yield and active ingredients.	Bajaj et al., 2014
Herbal medicinal plants	Increased vegetative growth, and Increased quality and quantity of herbal medicine	Das et al., 2012
*Linum album*	Biosynthesis of podophyllotoxin production	Kumar et al., 2013
*Stevia rebaudiana* and *Artemisia annua Chlorophytum borivilianum Spilanthes calva Withania somnifera*	Prominent leaf area and improved vegetative growth/yield early flowering in the crop and 90% survival on transplantation enhancement of the antifungal activity and quantity of spilanthol net primary productivity enhanced	Varma et al., 2013 Prasad et al., 2008 Rai et al., 2004; Prasad et al., 2008 Prasad et al., 2008
*Lantana camara*	The production of pentacyclic triterpenoids e.g., ursolic acid, oleanolic acid and betulinic acid	Kumar et al., 2015
*W. somnifera*	Stimulate plant growth and metabolism	Ahlawat et al., 2015

On the perspective of *P. indica* colonization role in crop plants, *P. indica*-mediated improvements in the growth and biomass have been reported in a number of crop plants including *Oryza sativa, Saccharum officinarum, Abrus precatorius, Zea mays, Phaseolus vulgaris*, and *Tridax procumbans* (Prasad, 2008; Varma et al., 2012a,b, 2013, 2014). *P. indica* can produce auxin (IAA) which in turn can promote plant root growth (Sirrenberg et al., 2007). In contrast to the auxin mediated least impact on the regulation of the gene expression in *Arabidopsis* (Vadassery et al., 2008), auxin regulated gene expression was found upregulated in *H. vulgare* (Schäfer et al., 2009) and Chinese cabbage (Lee et al., 2011). Interestingly, *P. indica* has been reported to interfere with ethylene signaling in plants where, it promotes the plant growth (Barazani et al., 2007). However, the exact mechanism underlying *P. indica*-mediated ethylene signaling inhibition is still unclear in plants (Hayat et al., 2010). The stimulation of secondary metabolite synthesis by the endophytic fungus, *P. indica* has also been reported for the production of pentacyclic triterpenoids (e.g., ursolic acid, oleanolic acid and betulinic acid) in the suspension cultures of *L. camara* (Kumar et al., 2015).

### Abiotic and Biotic Stress Tolerance

**FIGURE 3:**
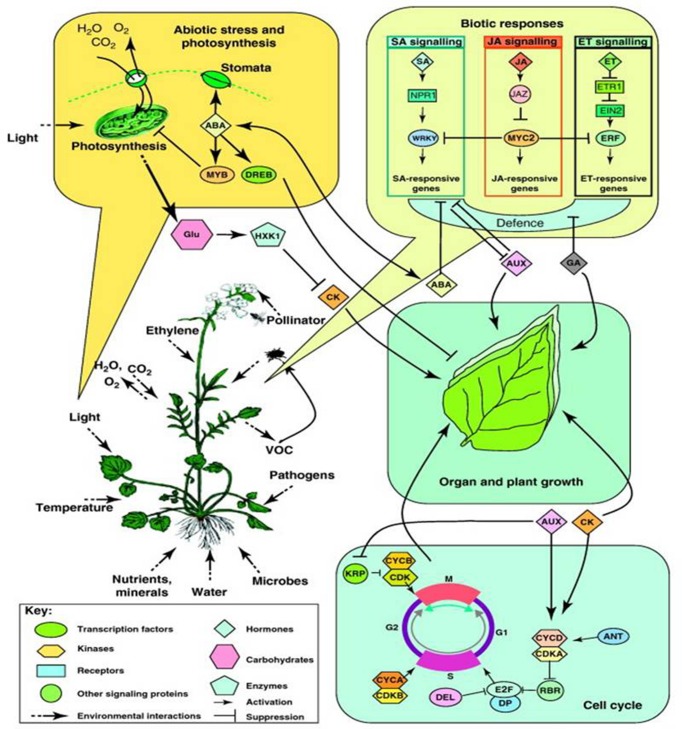
**Overview of biotic and abiotic stress responses in plants in nature**. A perceived stress factor induces changes at the cellular level (e.g., the cell cycle) that translate to the individual level (e.g., organ and plant growth; abiotic stress and photosynthesis) and influences interactions with other species (e.g., biotic responses). (Reprinted with permission from Keurentjes et al. (2011). Copyright Trends in Plant Science, Elsevier).

*Piriformospora indica* has been extensively reported to improve crop tolerance to a number of abiotic stresses including salinity, low temperature and heavy metal toxicity (Baltruschat et al., 2008; Sun et al., 2010; Husaini et al., 2012; Zarea et al., 2012; Ansari et al., 2013; Unnikumar et al., 2013). *P. indica* colonization-mediated high salinity tolerance was reported in *Triticum aestivum* (Zarea et al., 2012), that of drought stress tolerance in *Arabidopsis* seedlings (Sherameti et al., 2008), Chinese cabbage (Sun et al., 2010) and strawberry (Husaini et al., 2012). *P. indica* colonization has extensively been reported to mediate the activation of defense related genes (such as *PR*, *LOX2*, and *ERF1* genes) (Zarea et al., 2012), abiotic stress responsive genes (*DREB2A*, *CBL1*, *RD29A*) (Ansari et al., 2013) and osmoprotectants (proline, glycine betaine) (Waller et al., 2005; Trivedi et al., 2013) (**Figure 3**). The interaction of *P. indica* with *A. thaliana* roots is a unique model system to study symbiotic relationships. Recently, Vahabi et al. (2015) has reported a co-cultivation system which allowed them to investigate the effects of fungal exudates on the root transcriptome before and after the establishment of a physical contact, and during early phases of root colonization.

Mutualistic fungi *P. indica* has also evolved the ability to deliver molecules, called effectors, inside the cells to enhance microbial infection, and manipulate the host metabolism (Kloppholz et al., 2011; Plett et al., 2014). The effector protein is involved in the establishment and maintenance of symbiosis in endo- and ectomycorrhiza, allowing the fungus to manipulate the plant defense response (Kloppholz et al., 2011; Plett et al., 2011, 2014). Recently, Akum et al. (2015) reported the function of *P. indica* effector candidate PIIN_08944, a non-DELD effector, during the interaction of plants with *P. indica*. The authors noted the contribution of the candidate effector to plant colonization where the mutualistic fungus suppressed the salicylate -mediated basal resistance response (Akum et al., 2015). Effector protein, PIIN_08944 expression was detected during chlamydospore germination, and fungal deletion mutants (Pi_Δ_08944) showed delayed root colonization. PIIN_08944-expressing *A. thaliana* showed a reduced expression of flg22-induced marker genes of pattern-triggered immunity (PTI) and the SA-defense pathway. In *H. vulgare*, the expression of PIIN_08944 reduced the burst of reactive oxygen species (ROS) triggered by flg22. Therefore, PIIN_08944 was advocated to contribute to the root colonization by *P. indica* by interfering with SA-mediated basal immune responses of the host plant (Akum et al., 2015).

*Piriformospora indica* has also been reported to modulate major antioxidant defense enzymes monodehydroascorbate reductase and dehydroascorbate reductase (Vadassery et al., 2009; White and Torres, 2010; Foyer and Shigeoka, 2011; Hamilton et al., 2012) and the other components of ROS-scavenging system (Waller et al., 2005; Sun et al., 2010). *P. indica*-colonization was reported to mediate the up-regulation in a number of enzymes involved in ROS-metabolism in salinity exposed plants (Baltruschat et al., 2008). In addition, the establishment of ROS-scavenging system as a result of *P. indica* inoculation has been reported to confer plant tolerance to major abiotic stresses including salinity, drought, heat and heavy metal, and protection against fungal and viral pathogens (Waller et al., 2005; Serfling et al., 2007; Kumar et al., 2009; Sun et al., 2010; Ansari et al., 2013). Interestingly, down-regulation of genes encoding enzymes for ascorbate synthesis in *Arabidopsis* exhibited a greater colonization by this endophyte (Vadassery et al., 2009). However, a greenhouse study of ‘Hildares’ tomato (*Solanum lycopersicum* Mill.) inoculation with *P. indica* under osmotic stress and specific chloride toxicity conditions showed insignificant increases in the growth of *P. indica*-inoculated plants grown under severe salinity stress, and the responses corresponded a significant increase in CO_2_ assimilation rate in the final harvest and leaf water potential and transpiration rate in the first harvest (Al-Absi and Al-Ameiri, 2015). Though the mechanisms underlying *P. indica*-induced resistance has already been much explained for abiotic and biotic stress response in rhizobacteria-colonized plants little is known for mechanisms underpinning *P. indica*-induced resistance in abiotic and biotic stressed-plants (Van Wees et al., 2008).

*Piriformospora indica* has its well defined roles in the protection of plants against a range of biotic stress factors such as pathogenic fungi, bacteria and virus (Waller et al., 2005; Serfling et al., 2007; Oelmüller et al., 2009; Camehl et al., 2010, 2013; Molitor et al., 2011; Dolatabadi et al., 2012; Johnson et al., 2013). The analyses of the beneficial interaction between *P. indica* and host plants has revealed the upregulation of various defense related genes including pathogenesis related *PR* genes, jasmonate JA (*VSP*, *PDF1.2*, *LOX2*) and ethylene ET (*ERF1*) signaling genes in response to pathogen attack (Camehl et al., 2010; Molitor et al., 2011) (**Figure 3**). *P. indica* has found to confer protection to *H. vulgare* plant against root damage caused by *Fusarium culmorum* and against shoot infection with *Blumeria graminis* (Waller et al., 2005). Many root pathogens have been found to be directly inhibited by antagonistic activities of the endophytic fungus except *F. culmorum* (Waller et al., 2005), *P. herpotrichoides* (Serfling et al., 2007), *F. oxysporum* (Dolatabadi et al., 2012). To the other, systemically induced resistance by *P. indica* root-colonization has also been reported for many leaf pathogens. Recently, in root endophyte-colonized *H. vulgare* plants, a sub-set of defense-related genes was observed highly induced by leaf pathogens (Molitor et al., 2011). The role of *P. indica* in the protection of a number of plants against losses due to fungal pathogens infection has been summarized in **Table 1**.

*Piriformospora indica* has been credibly reported to defend crop plants against attack of viral as well as fungal pathogens and thereby providing them a better vegetative or generative development (Camehl et al., 2013). Recently, *P. indica* has been found to provide massive bioprotective potential to economically important different agricultural and horticultural crops against various plant pathogens and insect pests attack (Varma et al., 2012a). *P. indica* fungus could be utilized to increase fungal disease resistance in plants where, *P. indica*-infested crop plants were reported to exhibit their additional resistant against pathogenic fungi (Waller et al., 2005). *H. vulgare* plants subjected to the fungal pathogen such as *F. culmorum*, become free from root diseases when colonized with *P. indica*. Similarly, *P. indica* treated plants were also protected against root-pathogenic *Cochliobolus sativus* fungus (Oelmüller et al., 2009). *P. indica* fungus-mediated improved resistance of *H. vulgare* has been noted against the attack of root- and leaf-pathogens such as *Fusarium culmorum* and *Blumeria graminis* (Waller et al., 2005). Recently, *P. indica* colonized *H. vulgare* roots incited systemic resistance against the biotrophic leaf pathogen *B. graminis* f. sp. *hordei* (Molitor et al., 2011). *P. indica* was reported to be an effective candidate in biocontrol of take-all diseases of *T. aestivum* (such as *Gaeumannomyces graminis* var. *tritici*) (Ghahfarokhi and Goltapeh, 2010) and *Fusarium* wilt disease of lentil (Dolatabadi et al., 2012). Greenhouse experiments on *P. indica*-colonization with winter *T. aestivum*’s roots were resulted into significant reduced of incidence of leaf, stem, and root diseass caused by *B. graminis* f. sp. *tritici*, *Pseudocercosporella herpotrichoides* and *F. culmorum*. In a field trial, *P. herpotrichoides* infection was considerably decreased in endophyte-colonized plants. Systemic resistance against *B. graminis* attack was induced in *P. indica*-colonized plants (Serfling et al., 2007). *P. indica*-induced powdery mildew (*Golovinomyces orontii*) resistance in *Arabidopsis* necessitated JA-signaling involving JA-regulated genes to confer *G. orontii* tolerance. Furthermore, fungus has been evidenced to require merely cytosolic and not nuclear localized *NPR1* to provoke systemic induced resistance in *Arabidopsis* (Stein et al., 2008). Recent reports have evidenced that *P. indica*-induced GA can positively modulate plant defense response in roots. It has similar potential to induce systemic resistance (ISR) like *Trichoderma* spp. Pathogens attack requires basic compatibility to attain with their host plants involving definite upstream regulatory genes. The key mechanism has been defined either in rhizobacteria-mediated ISR or by plant inoculation with *P. indica*. Nevertheless, the majority of expression studies regarding *P. indica*-host communication center on root physiology. Further, systemic stimulation of a small number of defense-related genes or proteins have been depicted based on more or less a similar mechanism (Shoresh et al., 2010; Camehl et al., 2013).

## *Piriformospora indica*-Interaction with other Microorganisms

*Piriformospora indica* interaction (antagonism and cooperation) with other microorganisms has been reported to improve plant protection against environmental stresses (Pham et al., 2004; Porras-Alfaro and Bayman, 2011). *P. indica* interacts with a diverse group of microorganisms such as *Sebacina vermifera*, *Pseudomonas fluorescens* (rhizobacteria), *Chlamydomonas reinhardtii*, *G. graminis*, and other soil fungi (i.e., *Aspergillus niger, A. sydowii* and *Rhizopus stolonifer*). *P. indica* invaded *H. vulgare* roots were reported resistant against *Fusarium* infections (Deshmukh et al., 2007). These authors provided evidences that pathogenesis-related (PR) proteins do not affect *P. indica*-mediated response to confer resistance against *Fusarium* infections. *P. indica* was found to diminish the severity of disease caused by *V. dahliae* (Fakhro et al., 2010). The interaction of *P. indica* with the Pepino mosaic virus (PepMV) was evaluated in hydroponically grown *L. esculentum* where, the authors observed 30% reduction on the disease severity by *V. dahliae* (Fakhro et al., 2010). The growth of pathogenic fungi such as *A. sydowii*, *R. stolonifer* and *A. niger* has been reported to be entirely obstructed by *P. indica*. However, stimulation was noticed in the growth of the alga such as *C. reinhardtii* when cultured with *P. indica*. Though *P. indica* interacts with diverse class of bryophyte including mosses and liverworts but no growth promotion has been reported as a result of the interaction (Pham et al., 2004).

Among the Sebacinales, *P. indica* shows its interaction with *S. vermifera* in addition to multinucleate *Rhizoctonia* (Schäfer and Kogel, 2009). The pure cultures of closely related species such as *P. indica* and *S. vermifera* were reported essential for the germination, growth, development and yield and herbivore resistance of *Nicotiana attenuate* (Barazani et al., 2005). It has been reported that *P. indica*, *S. vermifera* and *Trichoderma* species act as effective biocontrol agent for take-all diseases in *T. aestivum* (Ghahfarokhi and Goltapeh, 2010). *P. indica* has been reported to support the growth and development of *Azotobacter chroococcum*, *Azospirillum brasilensis*, and *Bradyrhizobium* sp.; however, *P. indica*-mediated inhibition was noted in *Pseudomonas fluorescens* (Malla and Pokhare, 2008). Interestingly, there is an evidence that *P. indica* subsist rod-shaped 1–1.5 μm long bacteria in its cytoplasm and is characterized as *Rhizobium radiobacter* (Sharma et al., 2008). In a field experiment, *P. indica*-colonized plants exhibited significantly reduced disease severity caused by *P. herpotrichoides* (Serfling et al., 2007). *P. indica* and *R. leguminosarum* inoculated *Phaseolus* bean along with vermicompost revealed enhanced length and dry weight of both root as well as shoot with respect to treatment either deficient in vermicompost or with single inoculation (Tuladhar et al., 2013). *P. indica* either alone or in an interaction with *S. vermifera*, *Trichoderma viride* and *T. harzianum* was found to be more effective in reducing the severity of *Fusarium* wilt disease of lenti (Dolatabadi et al., 2012).

The studies on 18S rRNA and 20S rRNA sequence identity have revealed the fact that *P. indica* is closed relatives to the *Rhizoctonia* group and *Sebacinaceae* (Basidiomycetes) (Singh et al., 2003). In adition, *P. indica* affinity with *Glomeromycota* members such as Glomerales, Diversisporales as well as Archeosporales has been deciphered through *P. indica* characterization *via* immunofluorescence, Western blot, enzyme-linked immunosorbent assay along with immuno-gold (Singh et al., 2003). In response to signals from *P. indica*, MATH protein, *LRR1, LRR2*, *PDK*, *OXII*, *MAPK* genes were upregulated in the roots of *A. thalina* prior to colonization (Vadassery et al., 2009). On the other hand, [Ca^2+^]_cyt_ either induced various signaling course of actions, or defense interrelated responses were suppressed by supplementary factors (e.g., effectors) liberated by the fungus. These signaling events may be useful to understand the interactions of other beneficial fungi associated with economically important diverse crops for possible biotechnological applications (Oelmüller et al., 2009; Varma et al., 2013).

## *Piriformospora indica* – Biotechnological Significance-Appraisal

### *P. indica* as a Bio-control Agent and Plant Stress Response Mediator

It is believed that *P. indica*-colonization results into the activation of antioxidant system, which in turn improves crop plant tolerance against abiotic as well as biotic stresses (Prasad et al., 2013). The bio-protection performance of *P. indica* in *T. aestivum* has been evidenced against *B. graminis* f. sp. *tritici*, *P. herpotrichoides* and *Fusarium culmorum* (Serfling et al., 2007), and in *Z. mays* against the root parasite *Fusarium verticillioides* (Kumar et al., 2009). The fungus colonized plants were less susceptible to *Alternaria alternate* and *Colletotrichum falcatu* compared to non-colonized plants (Varma et al., 2012a). The ability of *P. indica* to synthesize hydroxamic acids – a secondary metabolite has been reported, which functions like a natural pesticide (Varma et al., 2001). The significance of *P. indica* as a bio-fertilizer as well as a bio-control agent has been strongly advocated (Waller et al., 2005; Varma et al., 2012a,b). Now, *P. indica* has become a paramount important candidate in microbiological and biotechnological research revealing several positive consequences on diverse crop plants (Barazani and Baldwin, 2013).

Ethylene, a gaseous plant hormone is produced in the majority of plant cells and controls various aspects of plant growth and development. Its positive and/or negative consequences has already been realized on flower, fruit ripening and leaf epinasty and abscission, suppression of apical dominance, leaf senescence, PCD, root nodulation, seed dormancy, seed germination and responsiveness to environmental stress including pathogen attack (Sharafzadeh, 2012; Varma et al., 2012a,b). The beneficial interaction of *A. thaliana* roots with mutualistic root endophytic fungus *P. indica* has been reported to induce ethylene, a gaseous plant hormone (Khatabi et al., 2012). Ethylene contributed a significant role in retaining the stability between beneficial as well as non-beneficial traits *via* signaling components ETR1, EIN2 and EIN2/EIL1 in a *P. indica* mutualism with plant’s roots (Camehl et al., 2010). In another study, the *P. indica* induced methionine synthase activity was reported to facilitate methionine cycle of ethylene biosynthetic pathway (Peškan-Berghöfer et al., 2004) during its colonization with plant roots via immuno suppression explaining surprisingly broad host range of the fungus (Jacobs et al., 2011). In a DNA microarray study, expression analysis of gene of *P. indica* colonized *H. vulgare* roots depicted differentially expressed ethylene related genes (Schäfer et al., 2009). Ethylene was reported to positively modulate the *P. indica*-plant interaction *via* signal molecules of fungi as well as plant receptors at the root cells surface after landing the fungal spores to attain the desired compatibility. Interestingly, ethylene signal magnitude also plays an important part in the colonization of plant roots by *P. indica*; where, ethylene signaling either inhibits or promotes the growth of hyphae depending upon magnitude of signaling (Camehl et al., 2013). It is now clear that to establish the symbiosis, ethylene signaling network requires definite biochemical or genetic actions to sustain a communication across the symbionts as well as host plants to provide physiological benefits to each partner (Ansari et al., 2013).

### *P. indica* as a Regulator of Genes Involved in Plant Metabolism and Mineral Uptake

*Piriformospora indica* interaction studies in *Arabidopsis* and *H. vulgare* have provided molecular basis of the beneficial plant–microbe interaction (Sherameti et al., 2005; Achatz et al., 2010; Yadav et al., 2010; Ngwene et al., 2013; Pedersen et al., 2013). The fungus in a mutual interaction with plants has already been known to provide enhanced nitrate/nitrogen uptake (Sherameti et al., 2005; Yadav et al., 2010). The up-regulation of *Nia2* gene of NR and *SEX1* gene of starch degrading enzymes was reported to influence a substantial source and sink relation (Sherameti et al., 2005). The additional sinks were remained balanced via higher rates of CO_2_ assimilation in *H. vulgare* plants colonized by *P. indica* with respect to the subsequent controls (Achatz et al., 2010). The elevated N, P and K endogenous content were reported in chickpea as well as black lentil plants colonized with *P. indica* (Nautiyal et al., 2010). In contrast, Fe and Cu deficiencies in sugar cane plants were surpassed if inoculated with *P. indica* (Gosal et al., 2011). The expression profile of receptor kinase in roots of *Arabidopsis* and enhanced uptake of radio-labeled P was observed after colonization of the endophytic fungus (Shahollari et al., 2005). *P. indica*-mediated P-uptake and transport were evidenced to improve plant growth and development via their impact on various regulatory, structural and addition to energy transfer processes (Kumar et al., 2011). Further, reduced endogenous content of phosphate in *Z. mays* plants were detected upon its colonization by *P. indica* mutant where, a phosphate transporter was knocked out (Yadav et al., 2010; Ngwene et al., 2013).

### *P. indica* as a Regulator of Genes Involved in Plant Stress Resistance and Defense

*Piriformospora indica* colonization provides certain benefits to the host plant such as tolerance to high salt and drought, resistance against heavy metal toxicity and protection from pathogen attack (Unnikumar et al., 2013). *P. indica* colonized *T. aestivum* showed an optimum growth under rising concentrations of salt (Zarea et al., 2012). Plants such as *Arabidopsis*, Chinese cabbage and strawberry were recovered from drought stress when pre-inoculated with endophyte *P. indica* (Sherameti et al., 2008; Husaini et al., 2012). The expression profile of the drought responsive genes such as *DREB2A*, *ANAC072*, *CBL1* in addition to *RD29A* were positively modulated in the leaves of *P. indica*-colonized plants under drought stress exposure (Sun et al., 2010). The drought tolerance was exhibited as higher endogenous proline level which in turn led to increased tolerance to osmotic stress in endophyte colonized plants when compared to non-colonized one (Zarea et al., 2012). *H. vulgare* plants pre-inoculated with *P. indica* displayed augmented profile of enzymatic and non-enzymatic antioxidants levels resulting increased tolerant to drought and high salt (Baltruschat et al., 2008). The increased antioxidants level was evident by reduced dehydroascorbate level via dehydroascorbate conversion to ascorbate and elevated glutathione level (Waller et al., 2005). The stress tolerance response of *P. indica*-colonized plants was found to be associated with burst accumulation of plastid-localized proteins such as Ca^2+^-sensing regulator (CAS) in the leaves of colonized plants (Sun et al., 2010). The use of *P. indica* was found to be more effective in improving salt stress tolerance in crop plants that consequently has opened an innovative and promising application of this fungus in sustainable agriculture, especially in the areas affected by salinity (Zarea et al., 2013). The differential expression profile of abiotic stress responsive genes putatively engaged in various stress response in *P. indica*-colonized plants is required for in-depth interpretation of the mechanism functions via induction of osmoprotectants and heat shock proteins in plant cell (Waller et al., 2005; Vadassery et al., 2009; Zarea et al., 2013).

In *P. indica*-inoculated host plants, pathogenesis related *PR* genes, JA (*VSP*, *PDF1.2*, *LOX2*) and ethylene ET (*ERF1*) signaling genes were reported up-regulated in response to pathogen attack (Camehl et al., 2010; Molitor et al., 2011). *P. indica* mediated *H. vulgare* plant protection was first observed against *F. culmorum* and *B. graminis* in root and shoot part, respectively (Waller et al., 2005). Enhanced antioxidant defense system has been evidenced as a major factor involved in *P. indica* colonization mediated beneficial effects in *H. vulgare*, *T. aestivum* and *Z. mays* roots (Waller et al., 2005; Serfling et al., 2007; Kumar et al., 2009). It is also evident from mutant study that not nuclear but the cytosolic non-expressor of *PR*-genes 1 (*NPR1*) form is required by the fungus in order to induce systemic resistance (Stein et al., 2008). For this reason, the mechanisms involved in *P. indica*-induced resistance in plants is more or less similar to systemic resistance induced by plant growth-promoting rhizobacteria-colonized plants (Van Wees et al., 2008). The endophyte provides inducing systemic resistance against pathogenic fungi and also protects the plant against damages caused by the invading pathogens via direct antagonism (Johnson et al., 2013).

## Conclusion and Future Perspectives

*Piriformospora indica* is a mycorrhiza like endophytic fungus which exhibits its versatility for colonizing the plant species with direct manipulation of plant hormone signaling and induces both local and systemic resistance to several fungal and viral plant diseases through signal transduction. *P. indica* is multifunctional in providing its services such as nutrient uptake, disease resistance, stress tolerance and growth-promotion (Unnikumar et al., 2013). This fungus has become an outstanding tool for biological hardening during transplantation of micro-propagated plantlets. *P. indica*-infestation in a number of medicinal plants has been reported to stimulate the synthesis of valuable secondary metabolites (Bagde et al., 2010a; Prasad et al., 2013), therefore possess commercial and biotechnological importance. The comparison of the *P. indica* genome with other fungi has revealed its classical features related with biotrophism as well as saprotrophism (Deshmukh et al., 2006; Qiang et al., 2011). *P. indica* has also evolved highly effective colonization strategies in diverse plant species (Deshmukh et al., 2006; Schäfer and Kogel, 2009; Qiang et al., 2011). The significant number of reports accumulated during last one decade has confirmed the *P. indica*-mediated improvements in the growth and yield of various plants, which includes crop plants, horticultural and medicinal plants. These reports also support the role of *P. indica* in crop tolerance to a number of abiotic as well as biotic stresses. Therefore, *P. indica* can be used as: (i) bio-control agent and plant stress response mediator, (ii) regulator of genes involved in metabolism and mineral uptake, and (iii) regulator of genes involved in resistance and defense of plants. It needs elaboration of a few studies done on the role of nano(bio)technology in acheiving major insights into and benefits of plant-interaction with fungi/*P. indica* (Suman et al., 2010; Menezes et al., 2015; Pandey et al., 2015; Prasad et al., 2015b). Furthermore, in order to commercially exploit the potentialities of *P. indica* in improving biotic and abiotic stress tolerance in crop plants, in addition to establishing *P. indica* inoculum production conditions and its formulation and stability; and exploring the persistence of the fungus in the environment, the future research should focus on revealing: (i) the mechanisms underpinning broad compatibility in root symbiosis, (ii) functional analyses of the effector-like proteins, (iii) symbiosis determinants, and (iv) identification of novel symbiosis/pathogenicity genes.

## Author Contributions

SSG, RG, AJ, AV, NAA, NT, and EP developed the idea and wrote/finalized the MS. SSG, DT, MA, NAA, EP, RP, KKS, MWA, AAA made the figures and developed table and helped in writing. All authors read and approved the approved the final manuscript.

## Conflict of Interest Statement

The authors declare that the research was conducted in the absence of any commercial or financial relationships that could be construed as a potential conflict of interest.
